# Role of the thalamic nucleus reuniens in mediating interactions between the hippocampus and medial prefrontal cortex during spatial working memory

**DOI:** 10.3389/fnsys.2015.00029

**Published:** 2015-03-10

**Authors:** Amy L. Griffin

**Affiliations:** Department of Psychological and Brain Sciences, University of DelawareNewark, DE, USA

**Keywords:** reuniens, working memory, hippocampus, medial prefrontal cortex, oscillatory synchrony

## Abstract

Despite decades of research, the neural mechanisms of spatial working memory remain poorly understood. Although the dorsal hippocampus is known to be critical for memory-guided behavior, experimental evidence suggests that spatial working memory depends not only on the hippocampus itself, but also on the circuit comprised of the hippocampus and the medial prefrontal cortex (mPFC). Disruption of hippocampal-mPFC interactions may result in failed transfer of spatial and contextual information processed by the hippocampus to the circuitry in mPFC responsible for decision making and goal-directed behavior. Oscillatory synchrony between the hippocampus and mPFC has been shown to increase in tasks with high spatial working memory demand. However, the mechanisms and circuitry supporting hippocampal-mPFC interactions during these tasks is unknown. The midline thalamic nucleus reuniens (RE) is reciprocally connected to both the hippocampus and the mPFC and has been shown to be critical for a variety of working memory tasks. Therefore, it is likely that hippocampal-mPFC oscillatory synchrony is modulated by RE activity. This article will review the anatomical connections between the hippocampus, mPFC and RE along with the behavioral studies that have investigated the effects of RE disruption on working memory task performance. The article will conclude with suggestions for future directions aimed at identifying the specific role of the RE in regulating functional interactions between the hippocampus and the PFC and investigating the degree to which these interactions contribute to spatial working memory.

## Introduction

Working memory refers to the holding of mind of task-relevant information for use in goal-directed behavior (Baddeley, 1986). Delay tasks are used to assess working memory in a variety of species. For these tasks, a cue must be held in memory over a temporal gap before an appropriate response can be emitted. Although many attempts have been made to discover the neural circuitry responsible for working memory, there are still many unanswered questions about how the brain accomplishes this important and remarkable phenomenon. It is well established that the prefrontal cortex is crucial for working memory, however it has become clear that the prefrontal cortex does not act alone and that instead working memory processes involve functional interactions among multi-regional interconnected networks within the brain. This review will focus on one of the circuits likely to be crucial for working memory: the circuit that includes the prefrontal cortex, the hippocampus and the ventral midline thalamic nuclei: the reuniens (RE) and rhomboid nuclei (RH). First, the individual contributions of each component of this circuit will be described, followed by a description of the anatomical connectivity between these structures, evidence for functional interactions within the circuit during working memory performance, and finally future directions in the effort to discover the mechanisms within this circuit that give rise to our ability to use working memory.

## Role of the mPFC in Working Memory

Like the dorsolateral prefrontal cortex of the primate, the rodent medial prefrontal cortex (mPFC), is known to be crucial for “executive” functions such as decision making, goal-directed behavior, and working memory (Kolb, 1990; Goldman-Rakic, 1995; Petrides, 1995; Miller et al., 2002; Fuster, 2008; Kesner and Churchwell, 2011). The mPFC has been historically associated with working memory functions based on the wealth of data demonstrating that mPFC lesions disrupt working memory tasks (Kolb et al., 1994; Floresco et al., 1997; Wang and Cai, 2006, 2008). However, it has been suggested that the emergence of working memory impairments is secondary to deficits in planning and flexibility (Granon and Poucet, 1995; Ragozzino et al., 1999; Lacroix et al., 2002). Indeed, damage to or inactivation of the mPFC impairs performance in tasks that require the suppression of a learned response such as extinction of appetitive Pavlovian conditioning (Griffin and Berry, 2004), consolidation of fear extinction (Burgos-Robles et al., 2007), task rule switching (Dias and Aggleton, 2000; Rich and Shapiro, 2007), and attentional set shifting (Birrell and Brown, 2000). Nonetheless, the mPFC is certainly involved in working memory, among other complex cognitive functions.

Evidence from *in vivo* electrophysiology studies shows that single mPFC neurons exhibit a variety of behavioral correlates during navigation and working memory tasks. Because the mPFC is the recipient of direct hippocampal input (Jay and Witter, 1991), it was reasonable to conclude that mPFC neurons would show some spatial tuning like place cells of the hippocampus. However, there have been conflicting reports regarding the degree of spatial selectivity exhibited by mPFC neurons (Jung et al., 1998; Euston and McNaughton, 2006; Burton et al., 2009). Instead, the most robust feature of mPFC neurons is their sustained activity during tasks that require information to be held over a temporal gap. These “delay cells” were first characterized in the monkey (Fuster and Alexander, 1971; Kubota and Niki, 1971; Funahashi et al., 1989; Miller et al., 1996), but have also been observed to varying degrees in the rat (Jung et al., 1998; Chang et al., 2002; Baeg et al., 2003). This persistent firing of prefrontal neurons is cited as the neural signature of working memory processes (Goldman-Rakic, 1995; Miller et al., 2002). Evidence in support of this idea comes from studies that have shown that mPFC neurons that show elevated activity during the delay period are predictive of future behavior. One investigation showed that mPFC neural activity ~1 s prior to a lever press predicted the lever choice (Chang et al., 2000). Similarly, (Baeg et al., 2003) examined mPFC ensemble activity during the delay period of a spatial delayed alternation task. They found that the neuronal population decoded the past and future goal arm choice with increasing accuracy as the rat became more proficient at performing the working memory task. Later, this same group (Baeg et al., 2007) found that functional connectivity between mPFC neurons diminished during learning but remained stable across memory retention, supporting the notion that memories are represented by changes in synaptic strength within a distributed network of mPFC neurons.

## Role of the Hippocampus in Working Memory

Spatial working memory tasks have been an essential tool for developing rodent models of memory. In a typical working memory task, the spatial alternation task, rats are placed on an elevated T-maze and required to alternate between the left and right goal arms on each trial. The task relies on the rat’s ability to remember which goal arm was visited on the previous trial in order to correctly select the opposite goal arm. There are two main versions of this task: continuous alternation (CA), in which the rat alternatively visits the left and right goal arms in a “figure 8” pattern, and delayed alternation (DA), in which the rat also alternates visits to the left and right goal arm, but pauses in the start box between trials. Due to the insertion of the delay period, during which the rat must remember the previously-rewarded goal location, the working memory demand is greater for DA than for CA. Accordingly, hippocampal lesions (or disruption of its inputs) lead to performance impairments in DA (Rawlins and Olton, 1982; Brito et al., 1983; Stanton et al., 1984; Czerniawski et al., 2009), but not CA (Ainge et al., 2007a). Deficits have also been seen in non-spatial working memory tasks (Olton and Feustle, 1981; Wood et al., 1999).

The lesion studies described above are supported by evidence of working memory coding by hippocampus neurons. Ainge et al. (2007b) reported that place fields in the start box, where the rat waited between trials, showed discriminative firing rates depending on the upcoming trial. Similarly, Pastalkova et al. (2008) examined hippocampal single-neuron activity during the delay period of a spatial alternation task and demonstrated that the population of recorded neurons fired in a unique sequence for left and right trials, with the sequence predicting the upcoming choice of the rat. Our laboratory has shown that during the delay period of the working memory-dependent DA task, a large population of dorsal hippocampal neurons exhibit discriminative firing rates depending on whether the upcoming trajectory led to the right or left goal arm. This same population of neurons did not show discriminative firing during the CD task (Hallock and Griffin, 2013).

## Hippocampal-mPFC Interactions in Working Memory

The link between hippocampal-mPFC oscillatory synchrony and working memory has been reviewed previously (Colgin, 2011; Gordon, 2011). Briefly, tasks or portions of a task with a working memory requirement are associated with increased hippocampal-mPFC synchrony. Manipulations that decrease this synchrony in turn impair working memory. For example, Sigurdsson et al. (2010) showed reduced hippocampal-mPFC coherence in a genetic mouse model of schizophrenia. This reduced synchrony is accompanied by working memory impairments.

A recent study (O’Neill et al., 2013) found that the ventral hippocampus is an important modulator of dorsal hippocampus-mPFC synchrony by demonstrating that inactivation of ventral hippocampus reduced dorsal hippocampus-mPFC coherence. It is still unclear, however, whether the role of the ventral hippocampus in hippocampus-mPFC synchrony is permissive or directive. Future studies will need to be done to answer this question.

One of the few laboratories that have combined mPFC lesions with hippocampal single unit recordings in freely moving animals (Kyd and Bilkey, 2003) found that prefrontal lesions decreased the stability of place field locations, due to an increased sensitivity to subtle changes in local cues in an otherwise unchanged environment. A more recent study also examined hippocampal-prefrontal interactions by performing lesions of the hippocampus and recording from mPFC neurons during a conditioned place preference task in which rats were required to wait in a goal zone before receiving food reward (Burton et al., 2009). Single mPFC neurons showed anticipatory activity during the wait time in the goal zone. Importantly, this activity was diminished in hippocampal-lesioned rats and this disruption was accompanied by impairments in task performance. This anticipatory activity likely represents the expectation of forthcoming events. The disruption of this activity in hippocampal-lesioned rats suggests that hippocampal input may provide the mPFC with contextual information that is necessary for the selection of appropriate responses.

Another approach that has been taken to show that the projection from hippocampus to mPFC is crucial for normal spatial working memory is using crossed unilateral inactivations or lesions of the hippocampus and mPFC. Muscimol is injected unilaterally into the intermediate or ventral hippocampus and into the contralateral mPFC. Because the hippocampal-prefrontal pathway is strictly ipsilateral (Ferino et al., 1987; Hoover and Vertes, 2007), crossed unilateral microinfusion of muscimol will disconnect the structures without completely inactivating the structures themselves. To control for muscimol volume, the control procedure is to inject muscimol unilaterally into the hippocampus and into the ipsilateral mPFC. Hippocampal-mPFC disconnection using the crossed unilateral lesion approach disrupts the performance of spatial working memory tasks (Floresco et al., 1997; Churchwell and Kesner, 2011). One unresolved issue in the working memory literature is the degree to which delay length determines the extent of mPFC and hippocampus involvement and cooperation. The prevailing view is that with increasing delay lengths, the mPFC and hippocampus, and particularly their interaction become more critical for performance. This view is supported by the finding that whether the hippocampus and mPFC operated in parallel or interacted was dependent upon the delay length of a spatial delayed non-match to sample working memory task. In the short-delay (10 s) version of the task, hippocampal-mPFC disconnection did not impair task performance, suggesting that the integrity of either region was sufficient for successful performance. However, when a longer delay (5 min) was imposed, hippocampal-mPFC disconnection resulted in a significant impairment (Churchwell and Kesner, 2011). These results suggest that there is a temporal constraint on hippocampal-prefrontal interactions during working memory.

## Anatomical Connectivity in the Hippocampus-RE-mPFC Circuit

The nucleus reuniens (RE) of the ventral midline thalamus is the largest of the midline thalamic nuclei and has been established as the major source of thalamic input to the hippocampus (Herkenham, 1978; Wouterlood et al., 1990; Bokor et al., 2002). Situated directly above the third ventricle (Van der Werf et al., 2002), the RE extends throughout the rostral-caudal extent of the thalamus (Bokor et al., 2002). The neighboring RH lies dorsal to the RE. The RE and RH are often grouped together in lesion studies, however there are subtle differences in their efferent and afferent connections, as will be discussed below. A large proportion of RE projection neurons are glutamatergic (Bokor et al., 2002). Wouterlood et al. (1990) showed that RE axons form asymmetrical (excitatory) synapses on CA1 and subiculum pyramidal cell distal dendrites in stratum lacunosum-moleculare. Consistent with this anatomical connectivity, RE stimulation produces strong excitatory effects at CA1 of the hippocampus (Dolleman-Van der Weel et al., 1997). In fact, Bertram and Zhang (1999) showed that excitatory actions of RE on CA1 were equivalent, if not greater than the excitatory actions of CA3 on CA1. RE selectively targets CA1 and subiculum, but avoids CA3 and DG, bypassing the trisynaptic loop. CA1 and subiculum in turn, send direct projections to ventral mPFC.

The laboratory of Robert Vertes has produced a series of studies that examined in detail the anatomical connectivity of the RE using retrograde and anterograde tracers. McKenna and Vertes (2004) showed that the RE receives extensive input from the entire dorsal-ventral extent of the mPFC, along with afferents from the subiculum, cortex, basal forebrain, dienchephalon, and brainstem. mPFC has four subdivisions (Berendse and Groenewegen, 1991; Ray and Price, 1992; Ongür and Price, 2000): the anterior cingulate cortex (ACC), the prelimbic (PL) region, the infralimbic region (IL), and the medial agranular cortex. Vertes (2002) injected Phaseolus vulgaris-leucoagglutinin (PHA-L) into all four subregions of the mPFC and found that all four subregions project densely to the RE. In a 2006 study, Vertes et al. (2006) used the anterograde tracer PHA-L to examine the efferent projections of RE and RH As had been previously shown, CA1 and subiculum of the hippocampal formation were heavily innervated by RE and RH. The RE/RH also projected heavily to PL, IL and ACC subregions of the mPFC. Compared to RE, RH projections were found to be more widespread, including the basolateral amygdala and the nucleus accumbens. (See also Vertes, 2006).

Although it was known that mPFC projects to RE and that RE projects to hippocampus, it was unknown whether the hippocampally-projecting RE neurons receive mPFC input. Vertes et al. (2007) used a combination of anterograde (PHA-L) and retrograde (Fluorogold; FLOR) tracers to visualize the synaptic connections of mPFC fibers on hippocampally-projecting RE neurons. Indeed, mPFC makes excitatory synaptic contact on the dendrites of hippocampus-projecting RE neurons. PHA-L injections in PL resulting in labeled fibers in RE and other midline thalamic nuclei. However, FLOR injections in ventral CA1 of hippocampus resulted in selective labeling in RE. This shows that prelimbic (PL) cortex projects to RE and other thalamic nuclei, but that RE is the only thalamic nucleus to project to CA1. RE neurons that project to hippocampus also receive inhibitory input on the soma, suggesting that inhibitory inputs to RE neurons can modulate RE output to hippocampus. Hoover and Vertes (2012) examined the degree to which RE neurons that project to hippocampus and mPFC are segregated or intermingled within the RE. They injected two separate retrograde tracers, one in hippocampus (ventral and dorsal divisions in separate animals) and one in mPFC. They found that RE projections to ventral hippocampus were generally more pronounced than to dorsal hippocampus. Rostral RE projected more strongly to hippocampus while caudal RE projected more strongly to mPFC. There was also a tendency for hippocampally-projecting neurons to be located in the medial aspect of RE and prefrontal-projecting neurons to be located in the lateral aspect of RE. Most interesting was that a significant proportion (3–9%) of RE neurons showed double labeling, indicating that these neurons project to both hippocampus and mPFC via axon collaterals. These results were subsequently confirmed, along with the demonstration that the subiculum region of the hippocampal formation contained a small population (~1%) of neurons that sends axons collaterals to both mPFC and RE (Varela et al., 2014).

Although the hippocampus sends a dense projection to mPFC, there are no return projections from the mPFC to the hippocampus (Goldman-Rakic, 1984; Room et al., 1985; Reep et al., 1987; Sesack et al., 1989; Hurley et al., 1991; Takagishi and Chiba, 1991; Buchanan et al., 1994; Hoover and Vertes, 2007). Together, the anatomical evidence shows that RE receives input from widely-distributed collection of limbic structures, but sends comparatively selective efferents out only to hippocampus, parahippocampus and the PFC. Thus, this region appears to be an important site of convergence and transfer of information between the hippocampus and mPFC.

## Role of the RE in Working Memory Tasks

Due to its anatomical connectivity with both hippocampus and mPFC, a number of investigations have targeted RE and the neighboring rhomboid nucleus (Rh) as a crucial region for working memory performance. Davoodi et al. (2009) were one of the first groups to specifically target the RE, examining the effects of RE inactivation on Morris water maze (MWM) performance. They reported both reference memory (RM) and working memory (working memory) impairments on MWM task performance after RE inactivation. First, it was shown that pretraining RE inactivation led to significant RM impairments on the acquisition stage of the task, but did not produce deficits in the 24-h memory retention test. Post-training and pre-probe test RE inactivation also impaired memory retrieval. Together, these results suggest that RE is necessary for RM acquisition, consolidation and retrieval in the MWM. For the working memory version of the task, the platform was moved to a new location and the rats were given an acquisition trial, followed 75 min later by a retrieval trial, in which they had to return to the new platform location. RE inactivation impaired working memory performance both when infusions were given before and after the acquisition trial. One confounding factor from this study was that working memory could not be distinguished from allocentric navigational performance. Deficits on the RM version of the task imply that RE inactivation interferes with the ability to use an allocentric spatial memory strategy, possibly due to indirect effects on hippocampal activity through silencing RE afferents to hippocampus. The working memory version of the task used a “delay” interval of 75 min, making it unlikely that the rat was using working memory rather than simply encoding the new arm position and retrieving the memory 75 min later. In other words, such a long delay interval violates the definition of working memory as a “holding in mind” of information because it is unlikely that the rats were holding the platform location in mind for such a long delay interval. The 75 min-delay version of the task is thus more likely to test long-term memory rather than working memory.

The RM deficits observed by Davoodi et al. (2009) were contradicted by another study. Dolleman-van der Weel et al. (2009) showed that RE excitotoxic lesions did not disrupt acquisition, retention or performance of the MWM task. Interestingly, the RE-lesioned rats performed better than controls when the task was switched to the visual platform variation. The authors attribute this superior performance to the loss of excitatory drive from RE to mPFC, which normally has an inhibitory influence on behavior. Thus, RE lesions did not impair acquisition of the RM variation of the MWM task and in fact, improved behavioral flexibility in this group. The RM version of the MWM is known to be dependent on hippocampal function, but independent of mPFC (Porter et al., 2000). In an effort to examine the involvement of RE/RH in a task that requires both hippocampal and mPFC function, Hembrook and Mair (2011) performed RE/RH lesions and examined performance in a radial arm maze task, which had been previously shown to depend on both hippocampus and mPFC (McDonald and White, 1993; Mair et al., 1998; Porter et al., 2000). In both the 8-arm version and the delayed response version of the task, RE/RH lesioned rats showed deficits compared to controls. In a follow-up study, Hembrook et al. (2012) then compared the effects of RE/RH inactivation on two different working memory tasks: a delayed-nonmatch-to-position (DNMTP) lever-pressing task and a varying choice delayed-nonmatch-to-place (VC-DNM) task in a radial arm maze. The DNMTP task had previously been shown to be dependent on both the hippocampus and the mPFC (Porter et al., 2000), whereas the VC-DNM requires hippocampal but not mPFC function. As predicted, RE inactivation produced deficits in the DNMTP, but not the VC-DNM task, supporting the notion that RE is required for tasks that involve both the hippocampus and the mPFC. Cholvin et al. (2013) provided additional support for this idea. This laboratory has designed a “double H maze” task that is thought to test both behavioral flexibility, a function usually associated with the mPFC and spatial memory, a function associated with the hippocampus (See Pol-Bodetto et al., 2011; Cassel et al., 2012). Similar to a previous study from the same laboratory (Loureiro et al., 2012), this study demonstrated that RE/RH inactivation did not disrupt recent memory retrieval in the MWM task. However, inactivation of the dorsal hippocampus, mPFC, and RE/RH all produced significant deficits in the double H maze task. The authors suggest that the double H maze task is sensitive to RE/RH inactivation because the task requires the use of both spatial memory and strategy shifting. Collectively, these studies show that the RE/RH are required for tasks that rely on the mPFC-hippocampal circuit and suggest that this region may be an important coordinator of hippocampal-mPFC interactions.

The task comparison approach utilized by the study just described is a powerful means of distinguishing between working memory processes and other non-mnemonic aspects of working memory tasks. However, the VC-DNM and DNMTP tasks are quite different in many respects. The DNMTP task is performed in an operant chamber and requires lever presses, whereas the VC-DNM task is performed on a radial arm maze, requiring navigation to the goal arms. To better dissociate working memory processes from motor, sensory, motivational and other potential confounding factors, our laboratory has developed a visuospatial conditional discrimination (CD) task that can be manipulated to be either dependent upon or independent of working memory. The two task variants are performed in the same testing room on the same T-maze. For both task variants, each trial consists of a maze traversal from the start box, down the maze stem to a one of the two goal arms, followed by a return to the start box, where the rat is confined for 20 s until the next trial. For both task variants, the rats must choose a goal arm based on a floor insert cue placed in the maze prior to the beginning of each trial. For example, rats learn that if the insert is covered with black mesh, they need to make a left goal arm choice in order to receive food reward and if the insert is smooth wood, they need to make a right goal arm choice. For the working memory variant of the task (CDWM), the floor inserts only cover the first two-thirds of the maze stem so that the cue is not available at the T-intersection of the maze, requiring that the rat hold the cue in memory for a brief period of time before making a right/left choice. For the standard CD task, the inserts cover the entire maze stem and goal arms, so that the cue is available at the time that the rat makes the goal arm choice. Our laboratory has shown that performance of the CD task is not impaired by PFC inactivation (Shaw et al., 2013) or dorsal hippocampal inactivation (Hallock et al., 2013a). In a recent study, we compared the effects of RE/RH inactivation between the CD and CDWM task. Separate groups of rats were trained on the two task variants until they reached asymptotic performance. After implantation of guide cannula aimed at the RE/RH, muscimol was used to temporarily inactivate the region immediately prior to testing. As predicted, RE/RH inactivation significantly impaired performance of the CDWM task, but had no effect on choice accuracy in the standard CD task (Hallock et al., 2013b). These results support the notion that RE is an important link between hippocampus and mPFC and that working memory processes rely on interactions within the hippocampus-RE-mPFC circuit.

## Conclusions

The evidence so far indicates that RE is crucial for working memory tasks, especially tasks that rely on both hippocampal and prefrontal function. Working memory may, however, be only one of many processes that involves the RE. Xu and Südhof (2013) found that the mPFC-RE-hippocampus circuit is critical for regulating fear memory generalization. Ito et al. (2013) have shown that RE neurons exhibit trajectory-specific coding similar to what has been observed in dorsal hippocampal neurons during a continuous alternation T-maze task (Wood et al., 2000). Head direction cells have also recently been found in the RE (Jankowski et al., 2014). Similar to hippocampus and prefrontal cortex, the RE may participate in a variety of memory-related behaviors. Functional interactions within this circuit however, may be specific to working memory. One future direction would be to compare hippocampal-mPFC functional interactions before and after RE inactivation to test the hypothesis that RE is a crucial modulator of hippocampus-mPFC synchrony during working memory. In conclusion, the electrophysiological, anatomical and behavioral results suggest that assigning a single behavioral correlate to RE/RH may be too restrictive. Instead if the role of the RE is to coordinate hippocampal-mPFC interactions (See Cassel et al., 2013), this region may be a crucial therapeutic target in the treatment of the cognitive deficits that accompany many of the major neuropsychiatric disorders.

## Conflict of Interest Statement

The author declares that the research was conducted in the absence of any commercial or financial relationships that could be construed as a potential conflict of interest.
